# *Photorhabdus africana* sp. nov. isolated from *Heterorhabditis* entomopathogenic nematodes

**DOI:** 10.1007/s00284-024-03744-3

**Published:** 2024-06-23

**Authors:** Ricardo A. R. Machado, Antoinette P. Malan, Anja Boss, Nicholle J. Claasen, Aashaq Hussain Bhat, Joaquín Abolafia

**Affiliations:** 1 Experimental Biology Research Group, Institute of Biology, University of Neuchâtel, Rue Emile-Argand 11, 2000 Neuchâtel, Switzerland; 2 Department of Conservation Ecology and Entomology, Stellenbosch University, Private Bag X1, Matieland, 7602 South Africa; 3 Department of Biosciences and University Center for Research and Development, Chandigarh University, Gharuan, Mohali, Punjab 140413 India; 4 Departamento de Biología Animal, Biología Vegetal y Ecología, Universidad de Jaén, Campus ‘Las Lagunillas’, Jaén, Spain; 5 Department of Biomaterials, Saveetha Dental College and Hospitals, Saveetha Institute of Medical and Technical Sciences (SIMATS), Saveetha University, Chennai, 600077 India

## Abstract

One Gram-negative, rod-shaped bacterial strain, isolated from an undescribed *Heterorhabditis* entomopathogenic nematode species was characterized to determine its taxonomic position. The 16S rRNA gene sequences indicate that it belongs to the class *Gammaproteobacteria*, to the family *Morganellaceae*, to the genus *Photorhabdus*, and likely represents a novel bacterial species. This strain, designated here as CRI-LC^T^, was therefore molecularly, biochemically, and morphologically characterized to describe the novel bacterial species. Phylogenetic reconstructions using 16S rRNA gene sequences show that CRI-LC^T^ is closely related to *P. laumondii* subsp*. laumondii* TT01^T^ and to *P. laumondii* subsp*. clarkei* BOJ-47^T^. The 16rRNA gene sequences between CRI-LC^T^ and *P. laumondii* subsp*. laumondii* TT01^T^ are 99.1% identical, and between CRI-LC^T^ and *P. laumondii* subsp*. clarkei* BOJ-47^T^ are 99.2% identical. Phylogenetic reconstructions using whole genome sequences show that CRI-LC^T^ is closely related to *P. laumondii* subsp*. laumondii* TT01^T^ and to *P. laumondii* subsp*. clarkei* BOJ-47^T^. Moreover, digital DNA-DNA hybridization (dDDH) values between CRI-LC^T^ and its two relative species *P. laumondii* subsp*. laumondii* TT01^T^ and *P. laumondii* subsp*. clarkei* BOJ-47^T^ are 65% and 63%, respectively. In addition, we observed that average nucleotide identity (ANI) values between CRI-LC^T^ and its two relative species *P. laumondii* subsp*. laumondii* TT01^T^ and *P. laumondii* subsp*. clarkei* BOJ-47^T^ are 95.8% and 95.5%, respectively. These values are below the 70% dDDH and the 95–96% ANI divergence thresholds that delimits prokaryotic species. Based on these genomic divergence values, and the phylogenomic separation, we conclude that CRI-LC^T^ represents a novel bacterial species, for which we propose the name *Photorhabdus africana* sp. nov. with CRI-LC^T^ (= CCM 9390^T^ = CCOS 2112^T^) as the type strain. The following biochemical tests allow to differentiate *P. africana* sp. nov. CRI-LC^T^ from other species of the genus, including its more closely related taxa: *β*-Galactosidase, citrate utilization, urease and tryptophan deaminase activities, indole and acetoin production, and glucose and inositol oxidation. Our study contributes to a better understanding of the taxonomy and biodiversity of this important bacterial group with great biotechnological and agricultural potential.

**Supplementary Information:**

The online version contains supplementary material available at 10.1007/s00284-024-03744-3.

## Introduction

Species of the bacterial genus *Photorhabdus* are symbiotically associated with *Heterorhabditis* entomopathogenic nematodes (EPNs) [1–4]. *Heterorhabditis* EPNs are soil-dwelling microorganisms that parasitize, kill and reproduce inside insects. These nematodes are cosmopolitan, and have been isolated from different locations around the globe, except Antarctica [5]. These nematodes establish a close symbiotic association with *Photorhabdus* bacteria. The nematodes carry the symbionts inside their intestines, and release them immediately after colonizing a host [6]. *Photorhabdus* bacteria produce toxins and digestive enzymes that kill and pre-digest the infected host [7–9]. Nematodes and bacteria then proliferate in the cadavers until all resources are depleted [10]. Subsequently, nematodes and bacteria re-establish symbiosis and abandon the cadaver in search of a new host [11]. These lethal organisms are important biological control agents and are broadly used to control agricultural pests [12–15]. In addition, *Photorhabdus* produces different bioactive compounds, including antibiotics, and therefore these organisms are of high biotechnological and medical relevance [16].

The type strain of the genus *Photorhabdus*, Hb^T^ (= ATCC 29999^T^), was isolated from *Heterorhabditis bacteriophora* entomopathogenic nematodes collected from Brecon (Australia), and was initially classified in the genus *Xenorhabdus*, together with other bacterial species isolated from the *Steinernema* entomopathogenic nematodes [17, 18]. To harmonize the taxonomy of the bacteria symbiotically associated with both genera of entomopathogenic nematodes, Boemare et al. (1993) proposed to create the genus *Photorhabdus* to accommodate the bacterial species associated with *Heterorhabditis* nematodes, and to maintain the genus *Xenorhabdus* for the bacterial species associated with *Steinernema* nematodes [19]. Since its creation, several *Photorhabdus* species and subspecies have been described [18–36]*.* Currently, the *Photorhabdus* genus contains 30 valid taxa: 23 species, 6 of which are divided into different subspecies. The species with validly published names are: *P. aegyptia, P. aballayi*, *P. akhurstii, P. antumapuensis, P. asymbiotica, P. australis, P. bodei*, *P. caribbeanensis*, *P. cinerea*, *P. hainanensis*, *P. heterorhabditis*, *P. hindustanensis*, *P. kayaii*, *P. khanii, P. kleinii*, *P. laumondii*, *P. luminescens*, *P. namnaonensis*, *P. noenieputensis*, *P. stackebrandtii*, *P. tasmaniensis, P. temperata*, and *P. thracensis*. Several species are in turn divided into different subspecies. *Photorhabdus akhurstii* is divided into *P. akhurstii* subsp. *akhurstii* and *P. akhurstii* subsp. *bharatensis*; *P. australis* is divided into *P. australis* subsp. *australis* and *P. australis* subsp. *thailandensis*; *P. heterorhabditis* is divided into *P. heterorhabditis* subsp. *heterorhabditis* and *P. heterorhabditis* subsp. *aluminescens*; *P. khanii* is divided into *P. khanii* subsp*. khanii* and *P. khanii* subsp*. guanajuatensis*; *P. laumondii* is divided into *P. laumondii* subsp. *laumondii* and *P. laumondii* subsp. *clarkei*; and *P. luminescens* is divided into *P. luminescens* subsp. *luminescens*, *P. luminescens* subsp. *mexicana*, and *P. luminescens* subsp. *venezuelensis* [34–37].

The aim of this study was to characterize a novel *Photorhabdus* bacterial species isolated from an undescribed *Heterorhabditis* nematode species collected in South Africa. For this, we biochemically, morphologically, and molecularly characterized one representative strain of this species, designed here CRI-LC^T^. We propose to name this novel bacterial species *Photorhabdus africana* sp. nov. Our study, therefore, contributes to a better understanding of the taxonomy and biodiversity of a bacterial group of biotechnological and agricultural relevance, and thereby further advance our efforts toward developing more biocontrol tools for sustainable and environmentally friendly agriculture.

## Materials and Methods

### Nematode Isolation and Identification

Entomopathogenic nematodes (EPNs) were recovered from soil samples using *Galleria mellonella* larvae as baits [38, 39]. Soil samples were collected from a citrus orchard located in the Sundays River Valley, Eastern Cape province, South Africa (GPS coordinates: 33°37′09.9″S, 25°40′23.7″E. Altitude: 30 m.a.s.l.). One of the nematode strains isolated was designated here as CRI-LC. It was identified as previously described [36]. Briefly, genomic DNA from about 20 females was extracted using the genomic DNA isolation kit from QIAamp DNA Mini Kit (Qiagen, Valencia, CA) following the manufacturer’s instructions. Two genes/genomic regions were amplified by polymerase chain reaction (PCR): the internal transcribed spacer (ITS) region of the rRNA gene and the cytochrome oxidase subunit I (COI) gene. To amplify the ITS region, the following primers were used: 18S (5′-TTGATTACGTCCCTGCCCTTT-3′) and 26S (5′-TTTCACTCGCCGTTACTAAGG-3′) [40]. To amplify the cytochrome oxidase subunit I (COI), primers LCO-1490 (5′-GGTCAACAAATCATAAAGATATTGG-3′) and HCO-2198 (5′-TAAACTTCAGGGTGACCAAAAAATCA-3′) were used [41]. PCR reactions consisted of 12.5 µL of DreamTaq Green PCR Master Mix (Thermo Scientific), 0.5 µL of each forward and reverse primers at 10 µM, 1 µL of genomic DNA and 10.5 µL of nuclease-free distilled water. The PCR reactions were performed using a thermocycler with the following settings. For the ITS region: 1 cycle of 5 min at 94 °C followed by 40 cycles of 30 s at 94 °C, 30 s at 50 °C, 1 min 30 s at 72 °C, and by a single final elongation step at 72 °C for 10 min. For the COI gene, the PCR program was as follows: one cycle of 94 °C for 2 min, followed by 37 cycles of 94 °C for 30 s, 51 °C for 45 s, 72 °C for 2 min, and a final extension at 72 °C for 12 min. PCR was followed by electrophoresis (45 min, 100 V) of 10 µL of PCR products in a 1% TBA (Tris–boric acid–EDTA) buffered agarose gel stained with SYBR Safe DNA Gel Stain (Invitrogen, Carlsbad, California, USA). PCR products were purified using QIAquick PCR Purification Kit (Qiagen, Valencia, CA) and sequenced using forward and reverse primers by Sanger sequencing (Microsynth AG, Balgach, Switzerland). The obtained sequences were manually curated, trimmed, and deposited in the NCBI database under the accession numbers given in Table S1. To obtain ITS and COI gene sequences of nematodes that belong to all the currently described species of the genus *Heterorhabditis*, we searched the database of the National Center for Biotechnology Information (NCBI) using the Basic Local Alignment Search Tool (BLAST) and the accession numbers described previously [2, 36, 42]. The resulting sequences were used to reconstruct phylogenetic relationships by the Maximum Likelihood method based on the Kimura 2-parameter nucleotide substitution model [43]. To select the best substitution models, best-fit nucleotide substitution model analyses were carried out in MEGA 7 [44]. Sequences were aligned with MUSCLE (v3.8.31) [45]. The trees with the highest log likelihood are shown. The percentage of trees in which the associated taxa clustered together is shown next to the branches. Initial tree(s) for the heuristic search were obtained automatically by applying Neighbor–Join and BioNJ algorithms to a matrix of pairwise distances estimated using the Maximum Composite Likelihood (MCL) approach, and then selecting the topology with superior log likelihood value. The trees were drawn to scale, with branch lengths measured in the number of substitutions per site. Graphical representation and edition of the phylogenetic trees were performed with Interactive Tree of Life (v3.5.1) [46, 47].

### *Bacteria* Isolation

To isolate the bacterial symbionts associated with as CRI-LC nematodes, *Galleria mellonella* larvae (Lepidoptera: Pyralidae) were infested with 150 infective juveniles. Three to four days later, several insect cadavers were dissected with a blade. Insect internal organs were spread onto Lysogeny Broth (LB) agar plates (Sigma-Aldrich, Switzerland) and incubated at 28 °C for 48–96 h. Based on colony morphology features, *Photorhabdus*-like colonies (yellow-orange colonies, bioluminescent) were sub-cultured until monocultures were obtained. Bacterial colonies that did not resemble *Photorhabdus* were not further sub-cultured or subjected to further characterization. Based on 16S rRNA gene sequences, seven bacterial monocultures were confirmed to belong to the *Photorhabdus* genus and to be conspecific. One of them was named CRI-LC^T^, designated the type strain of the species, and subjected to further characterization as described below.

### *Bacteria* Molecular Characterization

To molecularly characterize CRI-LC^T^, phylogenetic relationships were reconstructed using 16S rRNA gene and whole genome sequences. In addition, sequence similarity scores were calculated as described below.

### 16S rRNA Gene Sequencing

16S rRNA gene sequences were obtained as described previously [36]. Briefly, genomic DNA was extracted and purified using the GenElute Bacterial Genomic DNA Kit (Sigma–Aldrich, Switzerland) following the manufacturer’s instructions, and then, the 16S rRNA gene was amplified by polymerase chain reaction (PCR) using the following universal primers: 27F (5'-AGAGTTTGATCMTGGCTCAG-3') and 1525R (5'-AAGGAGGTGWTCCARCC-3') and the following cycling conditions: 1 cycle at 94 °C for 10 min followed by 40 cycles at 94 °C for 60 s, 55 °C for 60 s, 72 °C for 60 s and a final extension at 72 °C for 5 min [48–50]. PCR products were separated by electrophoresis in a 1% TAE-agarose gel stained with GelRed nucleic acid gel stain (Biotium), gel-purified (QIAquick Gel Purification Kit, Qiagen) and sequenced by Sanger sequencing (Microsynth AG, Balgach, Switzerland). The obtained sequences were manually curated using Bioedit 7.2.5 [51]. In addition, 16S rRNA sequences were obtained directly from the whole genome sequences using the bacterial ribosomal RNA predictor Barrnap [52]. Phylogenetic relationships were reconstructed using the Maximum Likelihood method based on the Kimura 2-parameter model in MEGA7 as described above [44, 53, 54]. The accession numbers of the sequences used for these analyses are shown in Table S2.

### Whole Genome Sequencing

Genome sequences were obtained as described previously [42, 55]. Briefly, genomic DNA was extracted and purified using the GenElute Bacterial Genomic DNA Kit (Sigma-Aldrich, Switzerland) following the manufacturer’s instructions. The resulting DNA was used for library preparation using the TruSeq DNA PCR–Free LT Library Prep (FC-121-3003) kit. Indexed libraries were then pooled at equimolar concentrations and sequenced (2 × 150 bp) on an Illumina HiSeq 3000 instrument. Genomes were assembled using the Bactopia pipeline [56]. To this end, the raw Illumina reads were quality trimmed using Trimmomatic 0.39 [57]. The resulting reads were assembled with SPAdes 3.14.1 (k-mer sizes of 31, 51, 71, 91, and 111 bp) [58]. Scaffolds with a mean read–depth smaller than 20% of the median read—depth of the longer scaffolds (≥ 5000 bp) as well as scaffolds that were shorter than 200 bp were removed. Minor assembly errors were corrected using Pilon 1.22 [59]. Completeness and contamination of the assembled genomes were assessed using checkM v1.2.2 with default parameters [60].

### Core Genome-Based Phylogenetic Reconstructions and Sequence Comparisons

To reconstruct whole genome-based phylogenetic relationships, genomes were first aligned using Roary 3.13.0. Genes to be considered core had to be present in 85% of the genomes with an 85% protein identity. Obtained alignments were used to build phylogenetic trees using FastTree 2.1.10 based on the Generalized Time Reversible Model (GTR). Graphical representation and edition of the phylogenetic trees were performed with Interactive Tree of Life (v3.5.1) [46, 47]. Digital DNA–DNA hybridization (dDDH) scores were used to determine pairwise whole genome sequence similarities. These scores were calculated using the GBPD (Genome Blast Distance Phylogeny) method through the Genome-to-Genome Distance Calculator 2.1 and formula 2 of the Deutsche Sammlung von Mikroorganismen und Zellkulturen (DSMZ) web service (http://ggdc.dsmz.de) using default parameters [61–64]. Digital DNA-DNA hybridization (dDDH) values of 70% and 79% delimit species and subspecies boundaries, respectively [27, 61, 65]. Average nucleotide identify (ANI) values were calculated using FastANI [66]. The accession numbers of the sequences used for these analyses are shown in Table S2.

### Genomic Comparative Analyses

Genomic comparative analyses to annotate and determine the presence/absence of genes that are involved in antibiotic resistance or in the production of specialized metabolites were carried out by aligning draft genome assemblies against the comprehensive antibiotic resistance database (“CARD”) [67–72] and against the antibiotics and secondary metabolite analysis shell (antiSMASH) database [73, 74]. Genes that passed the threshold values (antibiotic resistance: ≥ 70% nucleotide identity and ≥ 50% coverage; antiSMASH: ≥ 50% nucleotide identity) were considered as present in the genome [73, 74]. Below this threshold, genes were considered absent or nonfunctional.

### Physiological, Biochemical and Morphological Characterization

To physiologically, biochemically, and morphologically characterize CRI-LC^T^, bacterial cultures from single primary form colonies were used. Bacterial primary forms were determined by examining colony consistency (mucoid), and bioluminescence and pigment production [75]. The selected colonies were further sub-cultured and maintained on Lysogeny Broth (LB) agar plates at 28–30 °C. Cell morphology was observed under a Kern transmitted light microscope at 1000 × magnification, with cells grown for two days at 28 °C on LB agar plates. The optimum temperature for bacterial growth was evaluated on LB plates at 18, 23, 28, 32, 37, and 42 °C. Growth in media with varying salt concentrations and pH levels was evaluated in 3 mL of LB medium using 14 mL Falcon tubes. Three NaCl concentrations were used: 1% (Regular LB medium), 2%, and 3% (w/v). Three pH levels were used: 5, 7, and 9. Each tube was inoculated with 0.3 mL (OD_600_ = 1) of an overnight bacterial culture, then incubated for 24 h at 28ºC and 180 rpm, and finally the OD_600_ was measured using a spectrophotometer. Four tubes per treatment were evaluated. Cytochrome oxidase production was tested on discs containing *N,N*-dimethyl-p-phenylenediamine oxalate and *α*-naphthol (Sigma-Aldrich, Switzerland). Catalase activity was determined by adding a drop of 10% (v/v) H_2_O_2_ into 50 µL of a 16 h-old liquid LB-bacterial culture. Biochemical characterization was carried out using the API20E system (bioMérieux, Inc. Durham, NC) according to the manufacturer’s instructions. To this end, bacteria were grown for 16 h at 28 °C in LB agar Petri plates. Then, one single colony was re-suspended in 5 mL of 0.85% (w/v) NaCl. The resulting bacterial solution was used to inoculate the different microtubes containing the biochemical tests. Samples were incubated at 28 °C. Results were evaluated after 24 h. Bioluminescence production was evaluated by making photographs of bioluminescence on 24 h-old LB agar-cultured bacteria using an Amersham Imager 600 instrument (Cytiva, US).

### Ecological Characterization

To evaluate the entomopathogenic potential, bacteria were cultured overnight in LB liquid medium. Then, the bacterial cultures were collected and their optical densities at 600 nm (OD_600_) were measured. All cultures were then diluted to reach an OD_600_ = 1. The resulting cultures were serially diluted to obtain bacterial solutions with an OD_600_ = 0.01. 10 µL of the resulting bacterial solutions were injected into third-instar *G. mellonella* larvae. Eight larvae per bacterial strain were injected (*n* = 8). Control insects were injected with pure LB. Mortality was evaluated every 12 h for 3 days.

## Results and Discussion

### Nematode Molecular Identification

**Fig. 1:**
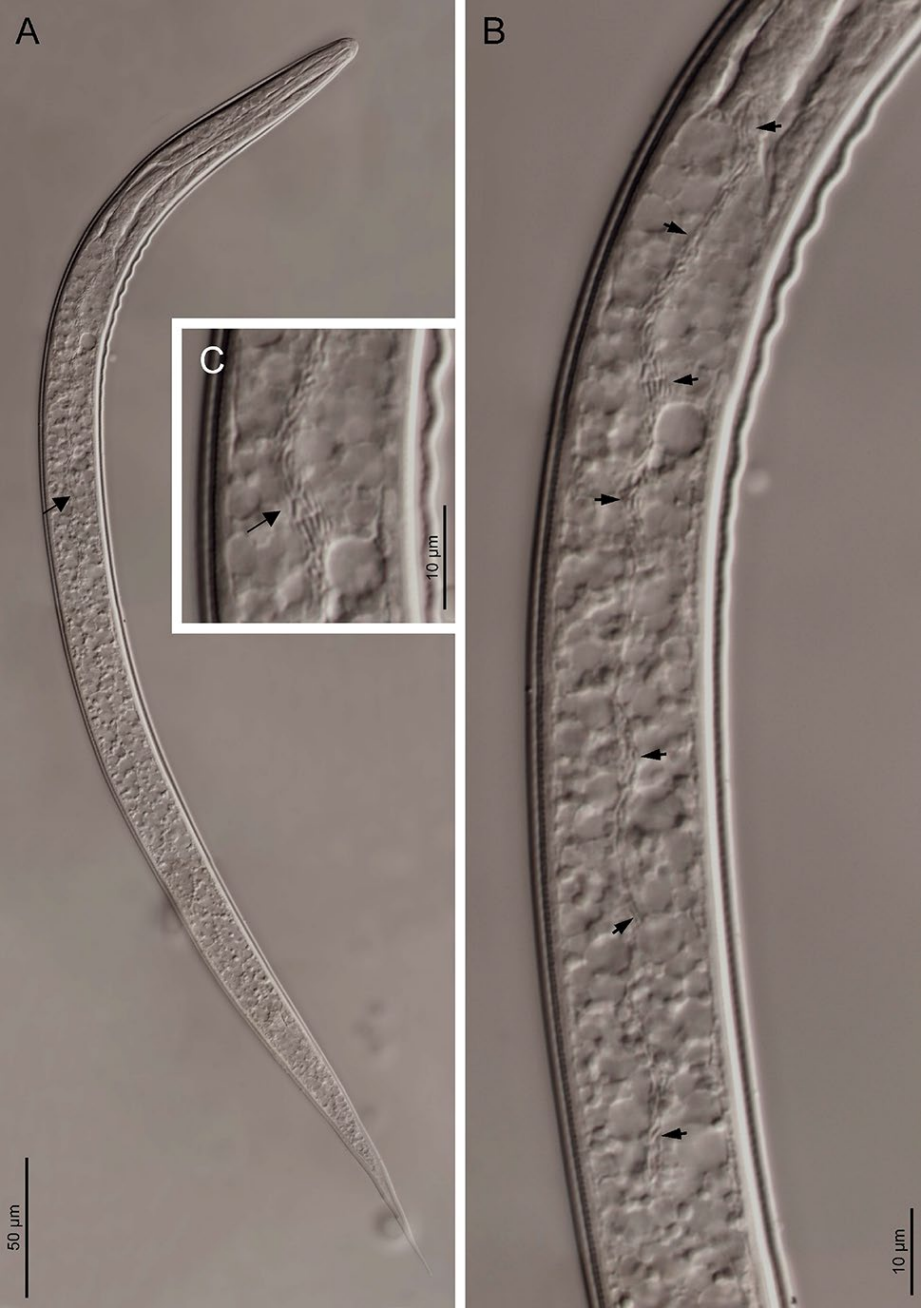
**A**–**C**) Light microscopy photographs of *Photorhabdus africana* sp. nov. CRI-LC^T^ in the second larval stage (L2) of *Heterorhabditis* sp. CRI-LC nematodes. Black arrows point to bacterial cells

**Fig. 2:**
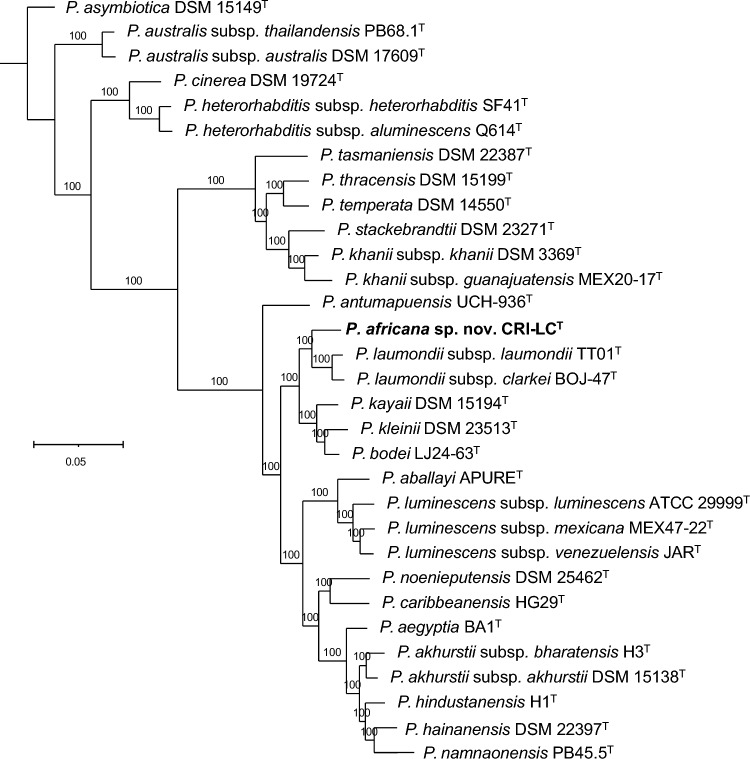
Phylogenetic reconstruction based on core genome sequences of *Photorhabdus* type strains with validly published names. 2237050 nucleotide positions (2228 core genes) were used in the analyses. Numbers at the nodes represent SH-like branch supports. Bar represents 0.05 nucleotide substitutions per sequence position. Accession numbers of the genome sequences used for the reconstruction are shown in Table S1

The bacterial strain, CRI-LC^T^, characterized in this study is hosted by entomopathogenic nematodes in their intestines (Fig. 1). Based on the sequences of the internal transcribed spacer (ITS) region of the rRNA gene and of the *COI* gene, these nematodes were found to belong to the *Heterorhabditis* genus, and likely represent a novel, undescribed species, closely related to *H. ruandica* and *H. zacatecana* (Fig. S1). Noteworthy, the symbiotic bacteria of *H. ruandica* nematodes is *P. laumondii* subsp. *laumondii* and the symbiotic bacteria of *H. zacatecana* nematodes is *P. kleinii* [42]. These two bacterial species are phylogenetically related to the symbiont of CRI-LC nematodes, showing certain degree of co-speciation (Fig. 2).

### 16S rRNA Gene-Based Phylogenetic Reconstruction and Sequence Comparisons

Phylogenetic reconstructions based on 16S rRNA gene sequences show that the bacterial strain isolated from *Heterorhabditis* CRI-LC nematodes, CRI-LC^T^, is closely related to *P. laumondii* subsp*. laumondii* TT01T and *P. laumondii* subsp*. clarkei* BOJ-47^T^ (Fig. S2). 16S rRNA gene sequence similarity scores between CRI-LC^T^ and these latest strains are 99.1% and 99.2%, respectively (Fig. S3). Using 16S rRNA and *gyrB* gene sequences as BLAST query, we found no records in the NCBI databank of other strains that could potentially be conspecific with CRI-LC^T^.

### Core Genome-Based Phylogenetic Reconstructions and Sequence Comparisons

**Fig. 3:**
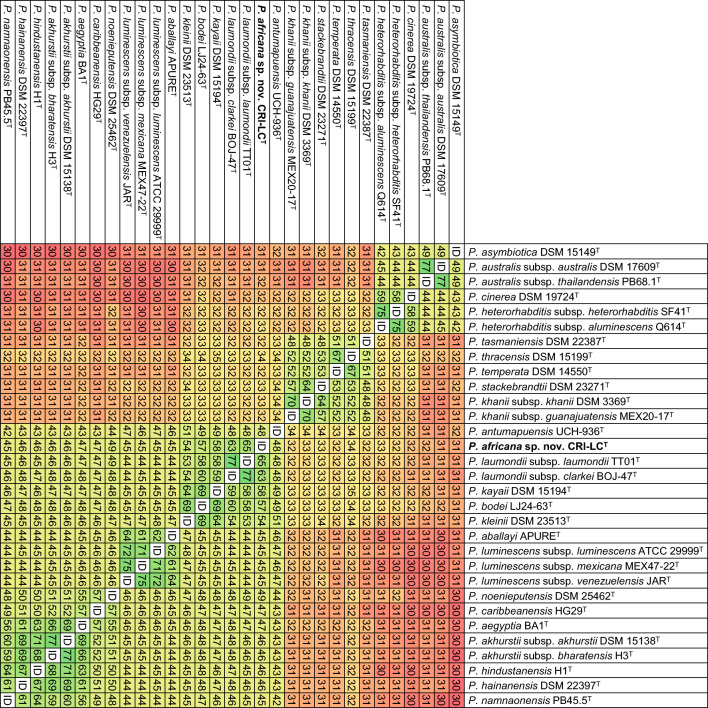
Pairwise comparison of digital DNA-DNA Hybridization (dDDH) scores (%) of *Photorhabdus* type strains with validly published names. Accession numbers of gene sequences used are shown in Table S1

16S rRNA gene-based phylogenetic reconstructions and sequence comparisons suggest that CRI-LC^T^ likely represents a new taxon, but they do not unambiguously resolve its phylogenetic relationships with other members of the genus or its taxonomic position. We therefore carried out a more detailed molecular characterization of CRI-LC^T^ using core genome-based phylogenetic reconstructions and by calculating whole genome sequence similarity scores [61–64, 66]. In core genome phylogenies, we observed that CRI-LC^T^ forms a distinct clade together with *P. laumondii* subsp*. laumondii* TT01^T^ and *P. laumondii* subsp*. clarkei* BOJ-47^T^ (Fig. 2). Due to the clear phylogenetic separations, CRI-LC^T^ appears to represent a novel taxon. To test this hypothesis, we calculated digital DNA-DNA hybridization (dDDH) and average nucleotide identity (ANI) scores using the GBPD (Genome Blast Distance Phylogeny) method and FastANI, respectively (Figs. 3, S4). We observed that dDDH scores between CRI-LC^T^ and *P. laumondii* subsp*. laumondii* TT01^T^, and between CRI-LC^T^ and *P. laumondii* subsp*. clarkei* BOJ-47^T^ are 65% and 63%, respectively. In addition, we observed that ANI values between CRI-LC^T^ and *P. laumondii* subsp*. laumondii* TT01^T^, and between CRI-LC^T^ and *P. laumondii* subsp*. clarkei* BOJ-47^T^ are 95.8% and 95.5%, respectively. These values are below the 70% dDDH and the 95–96% ANI divergence thresholds that delimits prokaryotic species (Figs. 2, S4) [61, 62, 66]. Based on these genomic divergence values and the phylogenomic separations, CRI-LC^T^ represents a new taxon, for which we propose the name *Photorhabdus africana* sp. nov. with CRI-LC^T^ (= CCM 9390^T^ = CCOS 2112^ T^) as the type strain.

## Genomic Features

### Genome Size, Nucleotide Composition, and Number of Predicted Coding Genes

The genome of *Photorhabdus africana* sp. nov. CRI-LC^T^ is predicted to contain 4560 protein-coding genes, a G + C content of 42.79%, and 5.2 Mbp (Table S3). These values are within the typical range observed for many of the species of the genus (Tables S3, S4). Using checkM (v1.2.2), the assembled genome is 96.42% complete, and has 2.29% of contamination.

### Predicted Antibiotic Resistance Phenotypes

In silico analyses allow to predict that *P. africana* sp. nov. CRI-LC^T^ and their most closely related species may be resistant to multiple antibiotics, which is a common trait in this bacterial genus (Tables S5). More specifically, the genome of *P. africana* sp. nov. CRI-LC^T^ contain genes that confer resistance to different antibiotics, but *P. africana* sp. nov. CRI-LC^T^ may be susceptible to elfamycins (Table S5).

### Predicted Biosynthetic Capacity

In silico analyses using the antibiotics and secondary metabolite analysis shell (antiSMASH) database uncovers the presence of biosynthetic gene clusters dedicated to the production of several polyketides and non-ribosomal peptides in the genome of *P. africana* sp. nov. CRI-LC^T^ and in the genomes of their more closely related taxa (Table S6). These metabolites are carotenoid, kolossin, luminmides, luminmycins, mevalagmapeptides, minimycins, odilorhabdins, piscibactins, putrebactins, ririwpeptides, syringopeptins, and tolaasins, which are typical compounds produced by this bacterial genus (Table S6). The production of kolossins, ririwpeptides, and syringopeptins differs between *Photorhabdus africana* sp. nov. CRI-LC^T^ and its more closely related species (Table S6).

### Physiological and Biochemical Characteristics

Biochemical tests show that *Photorhabdus africana* sp. nov. CRI-LC^T^ exhibits biochemical capacities that are similar to those of its more closely related species (Table 1). However, *P. africana* sp. nov. CRI-LC^T^ also exhibits unique biochemical capacities that differ from the biochemical capacities of their most closely related taxa, particularly, *β*-galactosidase, citrate utilization, urease and tryptophan deaminase activities, indole and acetoin production, and glucose and inositol oxidation (Table 1). Although, there are very few biochemical differences across the type strains of all the species/subspecies of this genus, additional biochemical tests potentially useful to differentiate the different taxa are presented in previous literature [26, 34]. Moreover, the three bacterial strains evaluated produce bioluminescence, which is a typical characteristic of this bacterial genus (Fig. S4).

**Table 1:** Phenotypic characters of: *Photorhabdus africana* sp. nov. CRI-LCT, *P. laumondii* subsp. *laumondii* TT01T, *P. laumondii* subsp. *clarkei* BOJ-47 T, P. *bodei* LJ24-63 T, and *P. namnaonensis* PB45.5 T

	CRI-LC^T^	TT01^T^	BOJ-47^T^	LJ24-63^T^	PB45.5^T^
*β*-Galactosidase	−	−	+	−	−
Arginine dihydrolase	−	−	−	−	−
Lysine decarboxylase	−	−	−	−	−
Ornithine decarboxylase	−	−	−	−	−
Citrate utilization	−	−	−	+	+
H_2_S production	−	−	−	−	−
Urease	−	+	−	−	−
Tryptophan deaminase	−	−	+	+	+
Indole production	−	+	−	+	V
Acetoin production	−	−	+	−	−
Gelatinase	+	+	+	+	+
Glucose oxidation	−	−	−	+	−
Mannitol oxidation	−	−	−	−	−
Inositol oxidation	+	−	−	−	−
Sorbitol oxidation	−	−	−	−	−
Rhamnose oxidation	−	−	−	−	−
Saccharose oxidation	−	−	−	−	−
Melibiose oxidation	−	−	−	−	−
Amygdalin oxidation	−	−	−	−	−
Arabinose oxidation	−	−	−	−	−
(Cytochrome) oxidase	−	−	−	−	−
No_2_ production	−	−	−	−	−
No_2_ reduction to N_2_ gas	−	−	−	−	−

### Ecological Characterization

When injected into the hemocoel of *G. mellonella* larvae, all the three bacterial strains rapidly killed the infected insects (Fig. S5). *Photorhabdus laumondii* subsp*. clarkei* BOJ-47^T^ was slightly more pathogenic than *P. africana* sp. nov. CRI-LC^T^, and *P. africana* sp. nov. CRI-LC^T^ was slightly more pathogenic than *P. laumondii* subsp*. laumondii* TT01^T^ within the first 24 h after infection (Fig. S5). However, all the three bacterial strains killed 100% of the infected insects within 48 h (Fig. S5).

## Protologue

### Description of Photorhabdus africana sp. nov.

(a.fri.ca'na. L. fem. adj. *africana* African, referring to the origin of the type strain). Cells are rod-shaped, approx. 2.0–2.2 µm wide and 4.0–5.1 µm long. Colonies are mucoid, circular, slightly irregular margins, yellow or orange in color, sometimes brownish (> 7-day-old cultures), with a diameter of approximately 1–2 mm after 48 h growth on LB agar. Produce bioluminescence. Bacterial growth in liquid LB occurs at temperatures between 18 °C and 37 °C. Optimal temperature for growth is 28–30 °C. Bacterial growth is strongly impaired at 37 °C, and no bacterial growth is observed at 42 °C. Bacteria grow in liquid LB with pH between 5 and 9 (optimum 7). Bacterial growth occurs in LB medium containing between 1% and 2% (w/v) NaCl (optimum 1%). Bacterial growth is inhibited in LB containing > 2% NaCl. Negative for cytochrome oxidase, *β*-galactosidase, arginine dihydrolase, lysine decarboxylase, ornithine decarboxylase, tryptophan deaminase, citrate utilization, urease, indole and acetoin production. Positive for gelatinase. Does not produce hydrogen sulfide. Oxidizes inositol. Does not oxidize glucose, mannitol, sorbitol, rhamnose, sucrose, melibiose, amygdalin or arabinose. Reduces nitrates. The type strain was isolated from an undescribed *Heterorhabditis* nematode species. Whole genome sequences of CRI-LC^T^ were deposited in the National Center for Biotechnology Information (NCBI) databank under the accession numbers JAXBVE01; and the 16S rRNA gene sequence under the accession numbers OR835571. The assembled genome contains 5200517 base pairs, 4560 proteins, and a 42.79% G + C content (Tables S2, S3). The type strain of the species, CRI-LC^T^, was deposited in the Czech Collection of Microorganisms (CCM) and in the national Culture Collection of Switzerland (CCOS) under the following accession numbers: CCM 9390^T^ and CCOS 2112^T^, respectively.

### Supplementary Information

Below is the link to the electronic supplementary material.

Supplementary file1 (PDF 624 KB)

## Data Availability

Whole genome sequences of CRI-LC^T^ were deposited in the National Center for Biotechnology Information (NCBI) databank under the accession numbers JAXBVE01; and the 16S rRNA gene sequence under the accession numbers OR835571.
