# Supplementary material for: Photorhabdus africana sp. nov. isolated from Heterorhabditis entomopathogenic nematodes
Source: Curr Microbiol. 2024 Jun 23;81(8):240. doi: 10.1007/s00284-024-03744-3 (PMC11194217; doi:10.1007/s00284-024-03744-3)
Supplement: Supplementary file 1 — Supplementary file1 (PDF 624 KB) [file 284_2024_3744_MOESM1_ESM.pdf]

-SUPPLEMENTARY MATERIAL-

Photorhabdus africana sp. nov. isolated from _Heterorhabditis_ entomopathogenic nematodes

Ricardo A. R. Machado[1]*, Antoinette P. Malan[2], Anja Boss[1], Nicholle J. Claasen[2], Aashaq H. Bhat[3], Joaquín Abolafia[4]

[1]*Experimental Biology Research Group. Institute of Biology. University of Neuchâtel. Neuchâtel, Switzerland.*

[2]*Department of Conservation Ecology and Entomology. Stellenbosch University. Private Bag X1, 7602 Matieland, South Africa.*

[3]*Department of Biosciences and University Center for Research and Development, Chandigarh University, Gharuan, Mohali, Punjab, 140413, India.*

[4]*Departamento de Biología Animal, Biología Vegetal y Ecología, Universidad de Jaén, Campus 'Las Lagunillas', Jaén, Spain.*

*Corresponding author: Ricardo A. R. Machado (ricardo.machado@unine.ch). Experimental Biology Research Group. Institute of Biology. University of Neuchâtel. Rue Emile-Argand 11, 2000 Neuchâtel, Switzerland. +41(0)327183076.

ORCID IDs: Machado: 0000-0002-7624-1105; Malan: 0000-0002-9257-0312

Whole genome sequences of CRI-LC^T were deposited in the National Center for Biotechnology Information (NCBI) databank under the accession numbers JAXBVE01; and the 16S rRNA gene sequence under the accession numbers OR835571.

-SUPPLEMENTARY FIGURES-

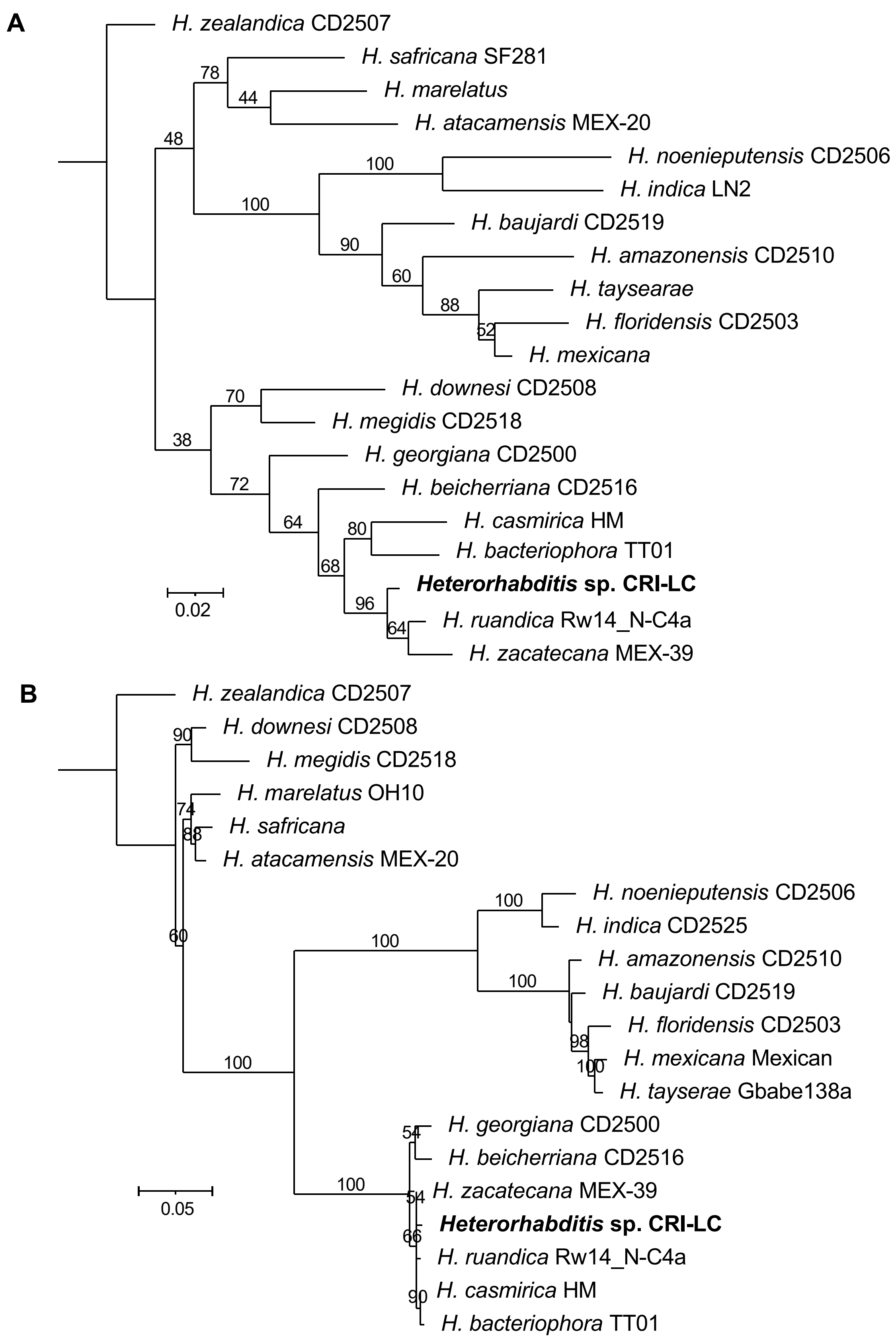

Figure S1. Maximum-likelihood phylogenetic trees based on: A) the cytochrome c oxidase I (*COI1*) and B) the ITS gene sequences of *Heterorhabditis* sp. CRI-LC, and of all the currently described species of the *Heterorhabditis* genus. The evolutionary histories were inferred by using the Maximum Likelihood method based on the Kimura 2-parameter model. The percentage of trees in which the associated taxa clustered together is shown next to the branches. The tree is drawn to scale, with branch lengths measured in the number of substitutions per site. Evolutionary analyses were conducted in MEGA7 based on 100 replications. Accession numbers of used gene sequences are shown in Table S1.

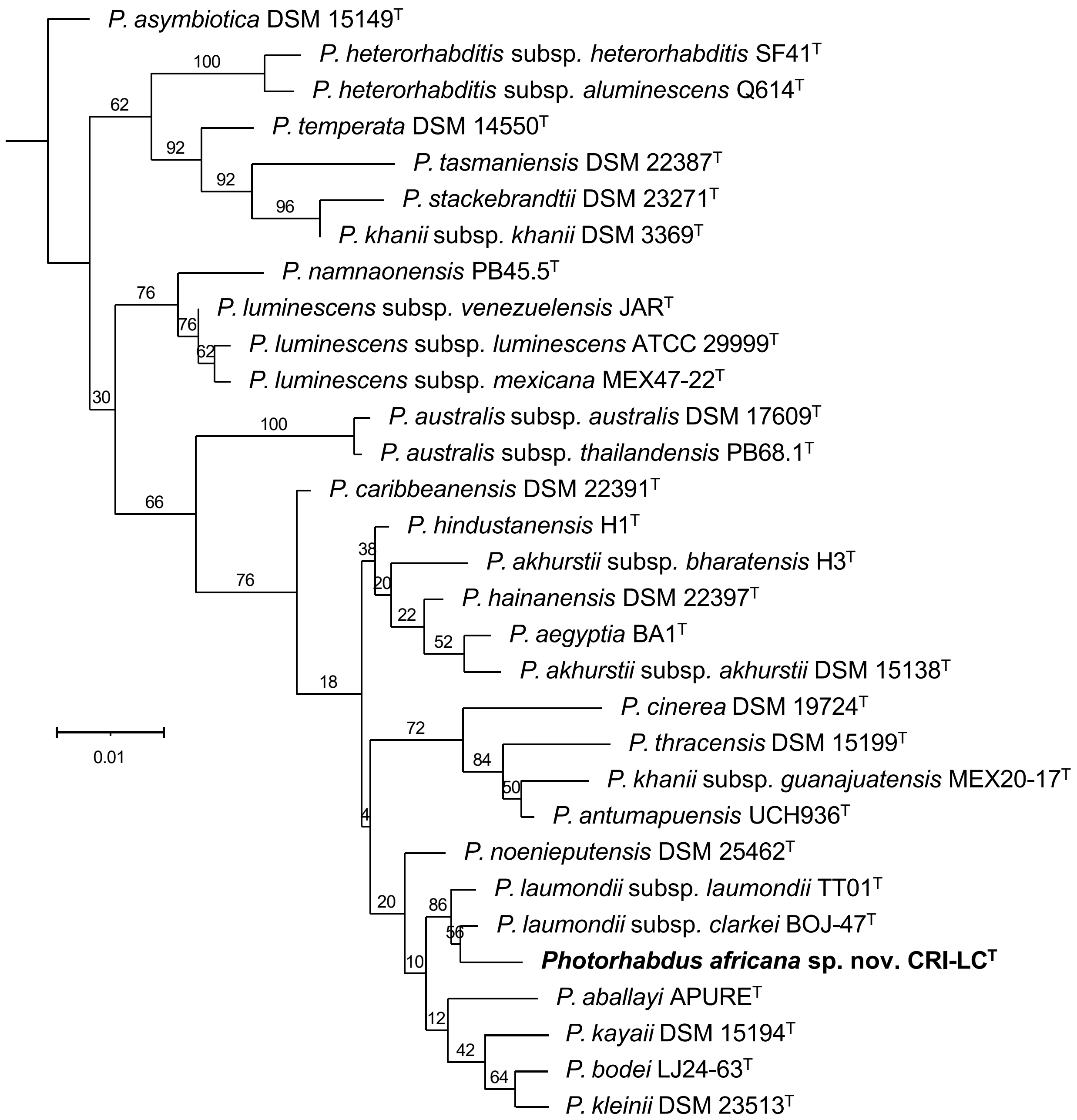

Figure S2. Maximum-likelihood phylogenetic tree reconstructed from 16S rRNA gene sequences of all *Photorhabdus* type strains with validly published names. The evolutionary history was inferred by using the Maximum Likelihood method based on the Kimura 2-parameter model. The tree with the highest log likelihood (-4273.14) is shown. The percentage of trees in which the associated taxa clustered together is shown next to the branches. The tree is drawn to scale, with branch lengths measured in the number of substitutions per site. There were a total of 1347 positions in the final dataset. Evolutionary analyses were conducted in MEGA7 based on 100 replications. Accession numbers of used gene sequences are shown in Table S2.

	P. asymbiotica DSM 15149^T	*P. heterorhabditis* subsp. *heterorhabditis* SF41^T	*P. heterorhabditis* subsp. *aluminescens* Q614^T	*P. temperata* DSM 14550^T	*P. tasmaniensis* DSM 22387^T	*P. stackebrandtii* DSM 23271^T	*P. khanii* subsp. *khanii* DSM 3369^T	*P. namnaonensis* PB45.5^T	*P. luminescens subsp. venezuelensis* JAR^T	*P. luminescens* subsp. *luminescens* ATCC 29999^T	*P. luminescens* subsp. *mexicana* MEX47-22^T	*P. australis* subsp. *australis* DSM 17609^T	*P. australis* subsp. *thailandensis* PB68.1^T	*P. caribbeanensis* HG29^T	*P. hindustanensis* H1^T	*P. akhurstii* subsp. *bharatensis* H3^T	*P. hainanensis* DSM 22397^T	*P. aegyptia* BA1^T	*P. akhurstii* subsp. *akhurstii* DSM 15138^T	*P. cinerea* DSM 19724^T	*P. thracensis* DSM 15199^T	*P. khanii* subsp. *guanajuatensis* MEX20-17^T	*P. antumapuensis* UCH-936^T	*P. noenieputensis* DSM 25462^T	*P. laumondii* subsp. *laumondii* TT01^T	*P. laumondii* subsp. *clarkei* BOJ-47^T	***P. africana* sp. nov. CRI-LC^T**	*P. aballayi* APURE^T	*P. kayaii* DSM 15194^T	*P. bodei* LJ24-63^T	*P. kleinii* DSM 23513^T
P. asymbiotica DSM 15149^T	ID	97.3	97.5	97.8	96.6	97.0	97.6	98.0	98.4	98.2	98.3	97.4	97.3	97.1	96.2	96.3	96.6	96.5	96.6	96.2	96.2	95.9	96.5	96.8	96.8	96.6	96.5	96.5	96.8	96.8	96.9
P. heterorhabditis subsp. *heterorhabditis* SF41^T	97.3	ID	99.3	97.5	96.5	96.8	97.3	97.0	97.2	97.0	97.2	97.0	97.1	96.5	95.7	95.8	95.9	95.8	95.9	96.7	96.2	96.4	96.2	96.0	96.2	95.9	95.7	95.9	95.3	95.7	95.6
P. heterorhabditis subsp. *aluminescens* Q614^T	97.5	99.3	ID	97.6	96.8	96.9	97.5	97.0	97.1	97.0	96.9	97.1	97.2	96.4	95.6	95.6	95.9	95.9	95.7	97.0	97.4	96.5	96.4	96.0	95.9	95.8	95.7	95.8	95.5	95.8	95.6
P. temperata DSM 14550^T	97.8	97.5	97.6	ID	97.6	98.1	98.4	97.6	98.1	97.9	97.9	96.5	96.6	96.9	96.2	96.2	96.8	96.5	96.3	97.0	97.4	97.0	97.4	96.6	96.3	96.3	96.5	96.6	96.2	96.8	96.5
P. tasmaniensis DSM 22387^T	96.6	96.5	96.8	97.6	ID	97.6	98.0	96.6	96.8	96.6	96.6	95.6	95.6	95.7	95.2	95.0	95.6	95.7	95.6	95.5	96.6	96.1	96.6	95.3	95.3	95.0	95.3	95.3	95.4	95.3	95.0
P. stackebrandtii DSM 23271^T	97.0	96.8	96.9	98.1	97.6	ID	99.4	96.8	96.8	96.9	97.0	95.9	95.8	96.5	95.9	96.0	96.5	95.9	95.9	96.3	97.4	97.6	98.1	96.5	96.1	96.0	96.1	96.0	95.8	96.2	96.2
P. khanii subsp. *khanii* DSM 3369^T	97.6	97.3	97.5	98.4	98.0	99.4	ID	96.9	97.1	97.2	97.3	96.3	96.2	96.5	95.7	95.7	96.2	95.9	95.9	96.2	97.4	97.3	97.8	96.2	96.1	95.6	95.8	95.9	95.7	95.9	95.9
P. namnaonensis PB45.5^T	98.0	97.0	97.0	97.6	96.6	96.8	96.9	ID	99.0	98.7	98.9	96.4	96.5	97.3	97.1	96.9	97.9	97.6	97.8	95.9	96.4	96.0	96.4	97.5	97.1	97.1	97.5	97.0	97.0	97.2	97.3
P. luminescens subsp. venezuelensis JAR^T	98.4	97.2	97.1	98.1	96.8	96.8	97.1	99.0	ID	99.7	99.7	96.9	97.0	97.5	97.1	97.1	97.8	97.3	97.3	96.1	96.5	96.1	96.5	97.3	97.2	97.2	97.3	97.8	97.3	97.3	97.3
P. luminescens subsp. *luminescens* ATCC 29999^T	98.2	97.0	97.0	97.9	96.6	96.9	97.2	98.7	99.7	ID	99.7	96.6	96.7	97.5	97.0	97.0	97.8	97.2	97.0	95.9	96.5	96.1	96.5	97.0	97.0	96.9	97.2	97.8	97.0	97.2	97.0
P. luminescens subsp. *mexicana* MEX47-22^T	98.3	97.2	96.9	97.9	96.6	97.0	97.3	98.9	99.7	99.7	ID	96.8	96.8	97.6	97.1	97.3	97.9	97.3	97.3	95.9	96.4	96.2	96.5	97.3	97.3	97.2	97.3	97.9	97.2	97.3	97.3
P. australis subsp. *australis* DSM 17609^T	97.4	97.0	97.1	96.5	95.6	95.9	96.3	96.4	96.9	96.6	96.8	ID	99.7	97.3	96.2	96.6	96.9	96.8	96.5	97.0	96.0	96.3	96.5	96.8	96.8	96.7	96.6	96.5	96.6	96.6	96.8
P. australis subsp. *thailandensis* PB68.1^T	97.3	97.1	97.2	96.6	95.6	95.8	96.2	96.5	97.0	96.7	96.8	99.7	ID	97.4	96.4	96.8	97.0	96.9	96.7	97.0	96.2	96.3	96.5	97.0	97.0	96.8	96.8	96.7	96.6	96.6	96.8
P. caribbeanensis HG29^T	97.1	96.5	96.4	96.9	95.7	96.5	96.5	97.3	97.5	97.5	97.6	97.3	97.4	ID	98.2	98.3	98.8	98.8	98.4	97.3	97.7	97.6	97.9	98.5	98.8	98.5	98.5	98.5	97.9	98.6	98.3
P. hindustanensis H1^T	96.2	95.7	95.6	96.2	95.2	95.9	95.7	97.1	97.1	97.0	97.1	96.2	96.4	98.2	ID	98.5	98.5	98.3	98.8	96.7	97.4	97.0	97.3	98.2	98.4	98.2	97.9	98.1	97.7	98.1	98.1
P. akhurstii subsp. *bharatensis* H3^T	96.3	95.8	95.6	96.2	95.0	96.0	95.7	96.9	97.1	97.0	97.3	96.6	96.8	98.3	98.5	ID	98.8	98.3	98.5	97.0	97.1	97.1	97.5	98.3	98.5	98.5	98.0	98.1	97.6	98.5	98.3
P. hainanensis DSM 22397^T	96.6	95.9	95.9	96.8	95.6	96.5	96.2	97.9	97.8	97.8	97.9	96.9	97.0	98.8	98.5	98.8	ID	99.2	98.8	97.1	97.9	97.5	97.9	98.5	98.5	98.3	98.6	98.6	98.2	98.8	98.6
P. aegyptia BA1^T	96.5	95.8	95.9	96.5	95.7	95.9	95.9	97.6	97.3	97.2	97.3	96.8	96.9	98.8	98.3	98.3	99.2	ID	99.1	97.2	97.9	97.3	97.6	98.4	98.6	98.3	98.5	98.5	98.3	98.9	98.5
P. akhurstii subsp. *akhurstii* DSM 15138^T	96.6	95.9	95.7	96.3	95.6	95.9	95.9	97.8	97.3	97.0	97.3	96.5	96.7	98.4	98.8	98.5	98.8	99.1	ID	97.1	97.8	97.0	97.4	98.4	98.5	98.2	98.2	98.1	97.9	98.5	98.2
P. cinerea DSM 19724^T	96.2	96.7	97.0	97.0	95.5	96.3	96.2	95.9	96.1	95.9	95.9	97.0	97.0	97.3	96.7	97.0	97.1	97.2	97.1	ID	97.3	98.1	98.0	97.3	97.0	97.1	97.0	97.3	97.1	97.3	97.2
P. thracensis DSM 15199^T	96.2	96.2	96.4	97.4	96.6	97.4	97.4	96.4	96.5	96.5	96.4	96.0	96.2	97.7	97.4	97.1	97.9	97.9	97.8	97.3	ID	98.2	98.7	97.5	97.4	97.0	97.0	97.6	97.1	97.6	97.3
P. khanii subsp. *guanajuatensis* MEX20-17^T	95.9	96.4	96.5	97.0	96.1	97.6	97.3	96.0	96.1	96.1	96.2	96.3	96.3	97.6	97.0	97.1	97.5	97.3	97.0	98.1	98.2	ID	99.1	97.7	97.3	97.2	97.0	97.6	97.0	97.6	97.4
P. antumapuensis UCH-936^T	96.5	96.2	96.4	97.4	96.6	98.1	97.8	96.4	96.5	96.5	96.5	96.5	96.5	97.9	97.3	97.5	97.9	97.6	97.4	98.0	98.7	99.1	ID	98.1	97.7	97.6	97.4	97.7	97.5	97.9	97.8
P. noenieputensis DSM 25462^T	96.8	96.0	96.0	96.6	95.3	96.5	96.2	97.5	97.3	97.0	97.3	96.8	97.0	98.5	98.2	98.3	98.5	98.4	98.4	97.3	97.5	97.7	98.1	ID	98.9	98.9	98.6	98.6	98.4	98.5	98.7
P. laumondii subsp. *laumondii* TT01^T	96.8	96.2	95.9	96.3	95.3	96.1	96.1	97.1	97.2	97.0	97.3	96.8	97.0	98.8	98.4	98.5	98.5	98.6	98.5	97.0	97.4	97.3	97.7	98.9	ID	99.5	99.1	98.6	98.4	98.5	98.6
P. laumondii subsp. *clarkei* BOJ-47^T	96.6	95.9	95.8	96.3	95.0	96.0	95.6	97.1	97.2	96.9	97.2	96.7	96.8	98.5	98.2	98.5	98.3	98.3	98.2	97.1	97.0	97.2	97.6	98.9	99.5	ID	99.2	98.5	98.5	98.6	98.8
***P. africana* sp. nov. CRI-LC^T**	96.5	95.7	95.7	96.5	95.3	96.1	95.8	97.5	97.3	97.2	97.3	96.6	96.8	98.5	97.9	98.0	98.6	98.5	98.2	97.0	97.0	97.0	97.4	98.6	99.1	99.2	ID	98.4	98.4	98.6	98.5
P. aballayi APURE^T	96.5	95.9	95.8	96.6	95.3	96.0	95.9	97.0	97.8	97.8	97.9	96.5	96.7	98.5	98.1	98.1	98.6	98.5	98.1	97.3	97.6	97.6	97.7	98.6	98.6	98.5	98.4	ID	98.6	98.6	98.2
P. kayaii DSM 15194^T	96.8	95.3	95.5	96.2	95.4	95.8	95.7	97.0	97.3	97.0	97.2	96.6	96.6	97.9	97.7	97.6	98.2	98.3	97.9	97.1	97.1	97.0	97.5	98.4	98.4	98.5	98.4	98.6	ID	98.8	98.8
P. bodei LJ24-63^T	96.8	95.7	95.8	96.8	95.3	96.2	95.9	97.2	97.3	97.2	97.3	96.6	96.6	98.6	98.1	98.5	98.8	98.9	98.5	97.3	97.6	97.6	97.9	98.5	98.5	98.6	98.6	98.6	98.8	ID	99.4
P. kleinii DSM 23513^T	96.9	95.6	95.6	96.5	95.0	96.2	95.9	97.3	97.3	97.0	97.3	96.8	96.8	98.3	98.1	98.3	98.6	98.5	98.2	97.2	97.3	97.4	97.8	98.7	98.6	98.8	98.5	98.2	98.8	99.4	ID

Figure S3. Pairwise nucleotide similarities (%) of 16S rRNA gene sequences of all *Photorhabdus* type strains with validly published names. 1347 nucleotide positions were analyzed. Accession numbers of used gene sequences are shown in Table S2.

	P. asymbiotica DSM 15149T	*P. australis* subsp. *australis* DSM 17609T	*P. australis* subsp. *thailandensis* PB68.1T	*P. cinerea* DSM 19724T	*P. heterorhabditis* subsp. *heterorhabditis* SF41T	*P. heterorhabditis* subsp. *aluminescens* Q614T	*P. tasmaniensis* DSM 22387T	*P. thracensis* DSM 15199T	*P. temperata* DSM 14550T	*P. stackebrandtii* DSM 23271T	*P. khanii* subsp. *khanii* DSM 3369T	*P. khanii* subsp. *guanajuatensis* MEX20-17T	*P. antumapuensis* UCH-936T	*P. africana* sp. nov. CRI-LCT	*P. laumondii* subsp. *laumondii* TT01T	*P. laumondii* subsp. *clarkei* BOJ-47T	*P. kayaii* DSM 15194T	*P. bodei* LJ24-63T	*P. kleinii* DSM 23513T	*P. aballayi* APURET	*P. luminescens* subsp. *luminescens* ATCC 29999T	*P. luminescens* subsp. *mexicana* MEX47-22T	*P. luminescens* subsp. *venezuelensis* JART	*P. noenieputensis* DSM 25462T	*P. caribbeanensis* HG29T	*P. aegyptia* BA1T	*P. akhurstii* subsp. *akhurstii* DSM 15138T	*P. akhurstii* subsp. *bharatensis* H3T	*P. hindustanensis* H1T	*P. hainanensis* DSM 22397T	*P. namnaonensis* PB45.5T
P. asymbiotica DSM 15149T	ID	92.5	92.4	90.7	90.6	90.5	85.5	85.9	85.6	85.9	85.7	85.8	85.9	85.7	85.6	85.5	85.6	85.7	85.6	85.5	85.6	85.6	85.6	85.4	85.3	85.5	85.4	85.3	85.3	85.2	85.1
P. australis subsp. *australis* DSM 17609T	92.5	ID	97.4	91.2	91.3	91.5	85.5	85.6	85.6	85.4	85.4	85.4	85.5	85.6	85.8	85.6	85.6	85.7	85.5	85.1	85.2	85.2	85.2	85.6	85.3	85.5	85.6	85.5	85.5	85.7	85.3
P. australis subsp. *thailandensis* PB68.1T	92.4	97.4	ID	91.2	91.1	91.2	85.5	85.7	85.5	85.5	85.4	85.3	85.5	85.6	85.7	85.6	85.6	85.7	85.4	85.1	85.2	85.2	85.2	85.6	85.4	85.7	85.7	85.6	85.6	85.7	85.6
P. cinerea DSM 19724T	90.8	91.2	91.2	ID	94.7	94.8	86.3	86.5	87.0	86.3	86.4	86.3	86.2	85.9	86.1	85.9	86.1	86.2	85.9	85.4	85.5	85.5	85.4	85.6	85.5	85.7	85.7	85.6	85.7	85.7	85.5
P. heterorhabditis subsp. *heterorhabditis* SF41T	90.6	91.3	91.2	94.7	ID	97.2	86.1	86.3	86.4	86.1	86.0	86.1	86.1	86.1	86.1	86.0	86.0	86.2	85.7	85.4	85.5	85.5	85.5	86.0	85.5	85.7	85.6	85.8	85.5	85.7	85.5
P. heterorhabditis subsp. *aluminescens* Q614T	90.6	91.5	91.3	94.8	97.3	ID	86.5	86.5	86.4	86.2	86.1	86.1	86.1	86.0	86.2	85.9	86.1	86.2	85.6	85.3	85.5	85.4	85.6	86.0	85.5	85.8	85.8	85.8	85.6	86.0	85.7
P. tasmaniensis DSM 22387T	85.4	85.4	85.4	86.2	86.1	86.4	ID	93.1	93.1	92.1	92.2	92.4	86.8	86.1	86.1	86.2	86.3	86.4	86.2	85.6	85.8	85.6	85.6	85.9	85.7	85.9	85.7	85.9	85.9	85.8	85.8
P. thracensis DSM 15199T	85.7	85.6	85.8	86.5	86.4	86.4	93.1	ID	95.8	93.3	93.2	93.3	87.0	86.7	86.4	86.6	87.2	86.9	86.8	85.9	86.0	85.9	85.9	86.4	85.9	86.2	86.1	86.2	86.1	86.2	86.1
P. temperata DSM 14550T	85.5	85.5	85.6	86.9	86.4	86.3	93.2	95.9	ID	93.1	93.2	93.3	86.9	86.3	86.2	86.3	86.5	86.5	86.3	85.8	86.0	85.8	85.9	86.0	85.9	86.1	86.1	86.0	86.1	86.0	85.9
P. stackebrandtii DSM 23271T	85.7	85.4	85.5	86.3	86.1	86.1	92.2	93.3	93.2	ID	95.9	94.4	87.4	86.4	86.3	86.2	86.5	86.5	86.7	86.0	86.1	86.1	86.4	86.1	85.9	86.0	85.8	86.0	85.8	85.8	85.9
P. khanii subsp. *khanii* DSM 3369T	85.7	85.4	85.3	86.3	85.9	86.1	92.2	93.2	93.1	95.9	ID	96.6	87.6	86.4	86.3	86.5	86.6	86.6	86.9	86.1	86.2	86.2	86.2	86.1	86.0	86.2	86.0	86.1	86.0	86.0	85.9
P. khanii subsp. *guanajuatensis* MEX20-17T	85.7	85.3	85.3	86.2	86.1	86.0	92.3	93.1	93.2	94.4	96.7	ID	87.6	86.2	86.3	86.3	86.5	86.6	86.8	86.1	86.1	86.3	86.1	86.2	85.9	86.2	86.0	86.1	86.1	85.9	85.9
P. antumapuensis UCH-936T	85.9	85.6	85.4	86.2	86.1	85.9	86.8	87.0	86.9	87.3	87.7	87.8	ID	92.4	92.3	92.3	92.6	92.6	93.1	91.9	92.0	91.9	92.2	91.3	91.1	91.2	90.9	91.0	91.0	90.9	90.8
P. africana sp. nov. CRI-LCT	85.7	85.8	85.7	86.1	86.3	86.2	86.2	86.6	86.4	86.5	86.5	86.4	92.4	ID	95.7	95.4	94.7	94.4	93.8	91.4	91.5	91.3	91.5	92.6	92.1	92.0	92.0	91.9	91.7	91.8	91.5
P. laumondii subsp. *laumondii* TT01T	85.7	85.8	85.8	86.1	86.2	86.2	86.1	86.5	86.2	86.3	86.3	86.3	92.2	95.8	ID	97.6	94.7	94.5	93.7	91.3	91.4	91.3	91.5	92.8	92.3	92.3	92.0	91.9	91.8	91.8	91.5
P. laumondii subsp. *clarkei* BOJ-47T	85.6	85.6	85.8	85.9	86.0	85.9	86.3	86.6	86.3	86.3	86.5	86.5	92.2	95.5	97.5	ID	94.7	94.9	93.8	91.3	91.4	91.4	91.5	92.6	92.3	92.5	92.5	92.5	92.4	92.2	91.9
P. kayaii DSM 15194T	85.7	85.7	85.8	86.3	86.2	86.1	86.5	87.2	86.6	86.5	86.6	86.6	92.5	94.7	94.7	94.8	ID	96.3	95.6	91.4	91.5	91.6	91.5	92.4	92.2	92.2	92.1	92.2	91.9	92.0	91.8
P. bodei LJ24-63T	85.7	85.8	85.8	86.4	86.3	86.3	86.5	86.9	86.8	86.5	86.6	86.5	92.5	94.4	94.5	94.8	96.3	ID	96.5	91.5	91.6	91.6	91.5	92.4	92.2	92.4	92.5	92.5	92.5	92.4	92.2
P. kleinii DSM 23513T	85.6	85.6	85.4	85.8	85.8	85.7	86.2	86.7	86.5	86.7	86.9	86.8	93.0	93.8	93.7	93.9	95.6	96.5	ID	91.9	92.3	92.2	92.3	92.1	92.0	92.1	91.9	91.9	91.8	91.8	91.5
P. aballayi APURET	85.4	85.2	85.1	85.4	85.3	85.3	85.6	85.9	85.8	86.0	86.1	86.2	91.8	91.3	91.3	91.3	91.4	91.4	92.0	ID	95.5	95.4	95.9	91.9	91.9	91.8	91.6	91.6	91.3	91.4	91.2
P. luminescens subsp. *luminescens* ATCC 29999T	85.4	85.2	85.2	85.5	85.5	85.5	85.9	86.0	86.1	86.0	86.2	86.3	92.0	91.4	91.3	91.3	91.5	91.5	92.2	95.5	ID	96.8	97.0	92.1	92.1	92.1	91.8	91.8	91.6	91.7	91.5
P. luminescens subsp. *mexicana* MEX47-22T	85.4	85.2	85.1	85.5	85.4	85.3	85.7	85.9	85.8	85.9	86.3	86.2	91.8	91.4	91.3	91.4	91.4	91.4	92.1	95.4	96.9	ID	97.3	92.2	92.0	91.9	91.7	91.8	91.6	91.6	91.4
P. luminescens subsp. *venezuelensis* JART	85.5	85.3	85.2	85.5	85.5	85.4	85.7	86.0	85.8	86.3	86.2	86.3	92.1	91.4	91.4	91.4	91.5	91.5	92.2	95.9	97.1	97.3	ID	92.1	91.9	91.9	91.7	91.7	91.6	91.5	91.4
P. noenieputensis DSM 25462T	85.3	85.4	85.4	85.6	86.0	85.9	85.8	86.2	86.0	86.0	86.0	86.2	91.4	92.6	92.8	92.6	92.4	92.4	92.2	91.9	92.2	92.2	92.2	ID	94.7	94.2	93.5	93.5	93.2	93.2	92.8
P. caribbeanensis HG29T	85.2	85.2	85.3	85.6	85.5	85.4	85.5	85.9	85.8	85.8	85.9	86.0	91.0	92.2	92.3	92.3	92.1	92.2	91.9	91.9	92.0	92.0	91.9	94.7	ID	94.9	93.7	93.8	93.3	93.4	92.9
P. aegyptia BA1T	85.4	85.4	85.6	85.8	85.6	85.6	85.9	86.1	86.2	85.9	86.2	86.1	91.1	92.0	92.2	92.4	92.2	92.3	92.0	91.7	92.0	92.0	91.9	94.1	94.8	ID	96.6	96.3	95.8	95.5	94.5
P. akhurstii subsp. *akhurstii* DSM 15138T	85.2	85.4	85.4	85.7	85.6	85.6	85.7	86.1	85.9	85.8	86.0	86.0	90.9	91.8	91.9	92.4	92.0	92.4	91.9	91.5	91.7	91.7	91.5	93.4	93.6	96.6	ID	97.7	96.9	96.5	95.3
P. akhurstii subsp. *bharatensis* H3T	85.2	85.4	85.4	85.7	85.6	85.6	85.8	86.0	85.9	85.8	86.1	86.1	90.9	91.8	91.9	92.5	92.0	92.3	91.8	91.5	91.8	91.8	91.7	93.5	93.7	96.3	97.7	ID	96.6	96.7	95.1
P. hindustanensis H1T	85.0	85.3	85.4	85.7	85.4	85.4	85.9	86.0	86.0	85.8	86.0	86.0	91.0	91.7	91.9	92.4	91.9	92.3	91.7	91.4	91.6	91.7	91.5	93.1	93.2	95.8	96.9	96.6	ID	96.4	95.8
P. hainanensis DSM 22397T	85.1	85.6	85.5	85.7	85.6	85.7	85.9	86.0	86.1	85.8	86.0	86.0	90.8	91.8	91.7	92.2	91.9	92.2	91.8	91.4	91.7	91.6	91.6	93.2	93.4	95.4	96.7	96.7	96.4	ID	95.4
P. namnaonensis PB45.5T	85.1	85.4	85.3	85.5	85.4	85.6	85.9	86.0	86.0	85.8	85.9	85.9	90.8	91.5	91.4	91.9	91.6	92.0	91.5	91.2	91.4	91.4	91.4	92.6	92.8	94.5	95.3	95.1	95.8	95.4	ID

Figure S4. Pairwise comparisons of average nucleotide identity (ANI) values (%) of all *Photorhabdus* type strains with validly published names. Accession numbers of used gene sequences are shown in Table S2.

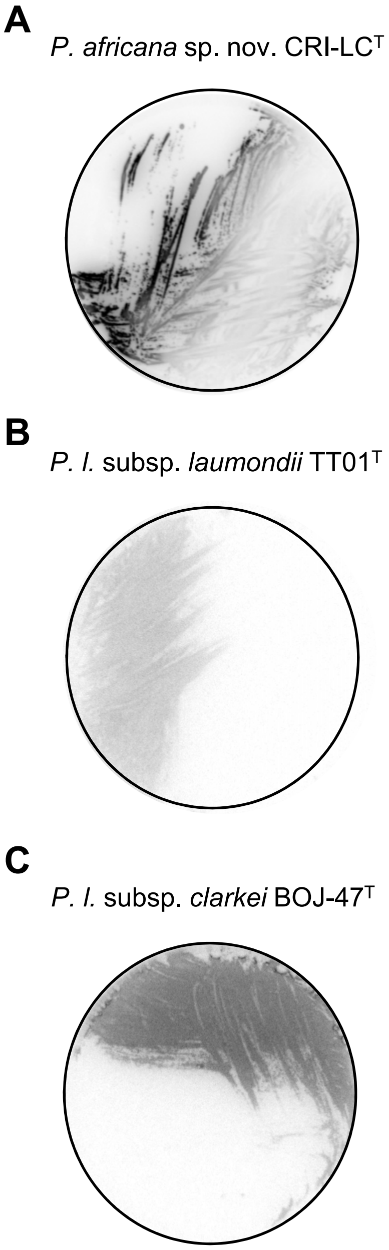

Figure S5. Production of bioluminescence by: A) *Photorhabdus africana* sp. nov. CRI-LC^{T}, B) *P. laumondii* subsp. *laumondii* TT01^{T}, and C) *P. laumondii* subsp. *clarkei* BOJ-47^{T}. Top-down photographs of 24h-old bacterial cultures grown in LB agar medium. Darker colours indicate higher bioluminescence levels. Photographs were made using an Amersham Imager 600 (Cytiva, US).

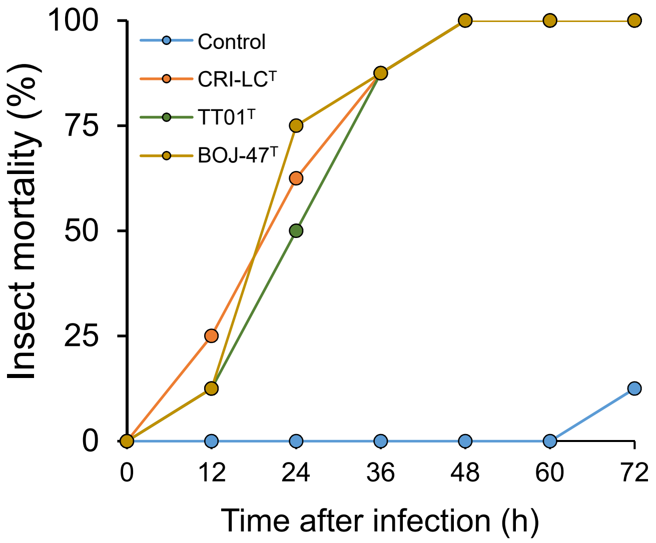

Figure S6. Entomopathogenicity of *Photorhabdus africana* sp. nov. CRI-LC^T, *P. laumondii* subsp. *laumondii* TT01^T, and *P. laumondii* subsp. *clarkei* BOJ-47^T. Insect mortality (%) caused by injecting the different bacterial strains at densities of OD_{600}=0.01.

-SUPPLEMENTARY TABLES-

Table S1. National Center for Biotechnology Information (NCBI) accession numbers of the nematode sequences used in this study. Sequences generated in this study are shown in bold.

Organism		Genetic Region	
**Species**	**Strain designation (s)**	**ITS**	**COI**
H. amazonensis	CD2510	MT372499	MT372499
H. atacamensis	MEX-20	MK421485	MW817979
H. bacteriophora	TT01	MZ326041	MW817975
H. baujardi	CD2519, F10	MT372500	MT373736
H. beicherriana	CD2516	MT372490	MT373730
H. casmirica	HM	OQ517969	OQ517936
H. downesi	CD2508, 23.9	MT372494	MT373732
H. floridensis	CD2503, K22	MT372501	MT373737
H. georgiana	CD2500	MT372492	MT373731
H. indica	CD2525	MT372498	-
	LN2	-	AB355853
H. marelatus	OH10	AY321479	-
	No information	-	EF043419
H. megidis	CD2518, DV	MT372495	MT373733
H. mexicana	Mexican	AY321478	-
	No information	-	EF043422
H. noenieputensis	CD2506, SF669	MT372497	MT373728
H. ruandica	Rw14_N-C4a	MZ326035	MW817969
H. safricana	No information	EF488006	-
	SF281	-	OR835850
H. taysearae	Gbabe138a	MF372596	-
	No information	-	EF043421
H. zacatecana	MEX-39	MZ326030	MW817964
H. zealandica	CD2507	MT372493	MT373734
Heterorhabditis sp.	CRI-LC	**OR852690**	**OR832295**

Table S2. National Center for Biotechnology Information (NCBI) accession numbers of the bacterial sequences used in this study. Sequences generated in this study are shown in bold.

Strain	16S	Genome
P. aballayi APURE^T	OP735347	JAPFCD01
***Photorhabdus africana* sp. nov. CRI-LC^T**	**OR835571**	**JAXBVE01**
P. aegyptia BA1^T	MT355495	JFGV01
P. akhurstii subsp. *akhurstii* DSM 15138^T	MK039086	RCWE01
P. akhurstii subsp. *bharatensis* H3^T	KJ995730	PUWU01
P. antumapuensis UCH-936^T	MZ676562	JAHZMK01
P. australis subsp. *thailandensis* PB68.1^T	MT355494	LOMY01
P. australis subsp. *australis* DSM 17609^T	AY280572	JONO01
P. asymbiotica ATCC 43949^T	Z76752	RBLJ01
P. bodei LJ24-63^T	MK039080	NSCM01
P. caribbeanensis DSM 22391^T	MK039083	RCWB01
P. cinerea DSM 19724^T	MK039069	PUJW01
P. hainanensis DSM 22397^T	MK039085	RCWD01
P. heterorhabditis subsp. *aluminescens* Q614^T	AY216500	JABBCS01
P. heterorhabditis subsp. *heterorhabditis* SF41^T	MK039068	RCWA01
P. hindustanensis H1^T	JX221722	PUWT01
P. kayaii DSM 15194^T	MK039081	JAJAFZ01
P. khanii subsp. *khanii* DSM 3369^T	MK039076	AYSJ01
P. khanii subsp. *guanajuatensis* MEX20-17^T	MK053912	PUJY01
P. kleinii DSM 23513^T	MK039079	JAJAFY01
P. laumondii subsp. *clarkei* BOJ-47^T	MK039078	NSCI01
P. laumondii subsp. *laumondii* TT01^T	MK039077	WSFH01
P. luminescens subsp. *luminescens* ATCC 29999^T	MK039082	FMWJ01
P. luminescens subsp. *mexicana* MEX47-22^T	MK053913	PUJX01
P. luminescens subsp. *venezuelensis* JAR^T	OP727818	JAPFFZ01
P. namnaonensis PB45.5^T	MK039087	LOIC01
P. noenieputensis DSM 25462^T	MK039084	RCWC01
P. stackebrandtii DSM 23271^T	MK039075	PUJV01
P. tasmaniensis DSM 22387^T	MK039072	PUJU01
P. temperata DSM 14550^T	MK039073	JAJAFX01
P. thracensis DSM 15199^T	MK039074	CP011104

Table S3. Features of the *Photorhabdus* genomes used in this study.

Scientific name	**Base pairs**	**Percent G+C**	**No. proteins**
P. aballayi APURE^{T}	5656459	42.42	4932
***P. africana* sp. nov. CRI-LC^{T}**	5200517	42.79	4560
P. aegyptia BA1^{T}	5004588	42.46	4259
P. akhurstii subsp. *akhurstii* DSM 15138^{T}	5552363	42.79	4799
P. akhurstii subsp. *bharatensis* H3^{T}	5514710	42.71	5043
P. antumapuensis UCH-936^{T}	5046591	42.52	4398
P. asymbiotica ATCC 43949^{T}	5103671	42.19	4397
P. australis subsp. *thailandensis* PB68.1^{T}	4800313	42.1	4301
P. australis subsp. *australis* DSM 17609^{T}	4912527	42.06	4436
P. bodei LJ24-63^{T}	5059726	42.71	4559
P. caribbeanensis DSM 22391^{T}	5362644	42.43	4651
P. cinerea DSM 19724^{T}	4899918	42.26	4613
P. hainanensis DSM 22397^{T}	5492441	42.83	4820
P. heterorhabditis subsp. *aluminescens* Q614^{T}	4981871	42.61	4660
P. heterorhabditis subsp. *heterorhabditis* SF41^{T}	5052136	42.24	4625
P. hindustanensis H1^{T}	5663704	42.86	5025
P. kayaii DSM 15194^{T}	5121090	42.54	4619
P. khanii subsp. *guanajuatensis* MEX20-17^{T}	5512522	43.52	4925
P. khanii subsp. *khanii* DSM 3369^{T}	5232343	43.54	4554
P. kleinii DSM 23513^{T}	5162879	42.41	4606
P. laumondii subsp. *clarkei* BOJ-47^{T}	5109397	42.52	4474
P. laumondii subsp. *laumondii* TT01^{T}	5376124	42.58	4721
P. luminescens subsp. *luminescens* ATCC 29999^{T}	5293870	42.6	4548
P. luminescens subsp. *mexicana* MEX47-22^{T}	5827786	42.53	5075
P. luminescens subsp. *venezuelensis* JAR^{T}	5429594	42.46	4585
P. namnaonensis PB45.5^{T}	5420031	42.7	4678
P. noenieputensis DSM 25462^{T}	5370579	42.56	4798
P. stackebrandtii DSM 23271^{T}	4846662	43.12	4427
P. tasmaniensis DSM 22387^{T}	5163014	43.56	4771
P. temperata DSM 14550^{T}	5382617	43.44	4872
P. thracensis DSM 15199^{T}	5147098	44.11	4542

Table S4. Features of the genomes of *Photorhabdus africana* sp. nov. CRI-LC^T generated in this study.

	**CRI-LC^T**
Scaffold L50	17
Scaffold N50	105357
Scaffold L90	55
Scaffold N90	25934
Scaffold len_max	221965
Scaffold len_min	202
Scaffold len_mean	14326
Scaffold len_median	470
Scaffold len_std	37066
Scaffold num_A	1487266
Scaffold num_T	1487911
Scaffold num_C	1119806
Scaffold num_G	1105534
Scaffold num_N	0
Scaffold num_bp	5200517
Scaffold num_bp_not_N	5200517
Scaffold num_seq	363
Scaffold GC content overall	42.79

Table S5. Antibiotic-resistance conferring genes of *Photorhabdus africana* sp. nov. CRI-LC^T, *P. laumondii* subsp. *laumondii* TT01^T, and *P. laumondii* subsp. *clarkei* BOJ-47^T. (+): present; (-): absent or non-functional.

Gene	Resistance mechanism	AMR Gene Family	Drug Class	CRI-LC^T	TT01^T	BOJ-47^T
CRP	Antibiotic efflux	Resistance-nodulation-cell division (RND) antibiotic efflux pump	Macrolides, fluoroquinolones, penam	+	+	+
rsmA			Fluoroquinolones, diaminopyrimidines, phenicols	+	+	+
KpnH		Major facilitator superfamily (MFS) antibiotic efflux pump	Macrolides, fluoroquinolones, aminoglycosides, carbapenems, cephalosporins, penams, peptides, penems	+	+	+
KpnF			Macrolides, aminoglycosides, cephalosporins, tetracycline, peptides, rifamycin.	+	+	+
qacJ		Small multidrug resistance (SMR) antibiotic efflux pump	Disinfecting agents and antiseptics	+	+	+
EF-Tu	Antibiotic target alteration	Elfamycin resistant EF-Tu	Elfamycins	-	+	-
PBP3		Penicillin-binding protein mutations conferring resistance to beta-lactam antibiotics	Cephalosporins, cephamycins, penams	+	+	+
ArnT		Phosphoethanolamine transferase	Peptide antibiotic	+	+	+

Table S6. Predicted specialized metabolites produced by *Photorhabdus africana* sp. nov. CRI-LC^T, *P. laumondii* subsp. *laumondii* TT01^T, and *P. laumondii* subsp. *clarkei* BOJ-47^T. (+): Produced; (-): Not produced.

Specialized metabolite	CRI-LC^T	TT01T	BOJ-47T
Carotenoid	+	+	+
Kolossin	-	+	+
Luminmides	+	+	-
Luminmycin A	+	+	+
Mevalagmapeptide	+	+	-
Minimycin	+	+	+
Odilorhabdins	+	+	+
Piscibactin	+	+	+
Putrebactin	+	+	+
Ririwpeptide	-	-	+
Syringopeptin	-	+	-
Tolaasin A	+	-	-
